# A qualitative study documenting unmet needs in the management of diabetic kidney disease (DKD) in the primary care setting

**DOI:** 10.1186/s12889-021-10959-7

**Published:** 2021-05-17

**Authors:** Manasi Datar, Saranya Ramakrishnan, Elizabeth Montgomery, Steven G. Coca, Joseph A. Vassalotti, Thomas Goss

**Affiliations:** 1 Boston Healthcare Associates, Boston, MA USA; 2 National Kidney Foundation, Inc., New York, NY USA; 3 Icahn School of Medicine at Mount Sinai, New York, NY USA

**Keywords:** Diabetic kidney disease, Type 2 diabetes, Primary care, Primary care provider, Inconsistent screening, Risk assessment, eGFR, Albuminuria

## Abstract

**Background:**

A majority of diabetic kidney disease (DKD) patients receive medical care in the primary care setting, making it an important opportunity to improve patient management. There is limited evidence evaluating whether primary care physicians (PCPs) are equipped to effectively manage these patients in routine clinical practice. The present study was undertaken to identify gaps in primary care and unmet needs in the diagnosis and monitoring of DKD in type 2 diabetes (T2D) patients among PCPs.

**Methods:**

This was a qualitative analysis based on 30–45-min interviews with PCPs treating T2D patients. PCPs were recruited via email and were board-certified, in practice for more than 3 years, spent most of their time in direct clinical care, and provided care for more than three T2D patients in a week. Descriptive data analysis was conducted to identify and examine themes that were generated by interviews. Two reviewers evaluated interview data to identify themes and developed consensus on the priority themes identified.

**Results:**

A total of 16 PCPs satisfying the inclusion criteria were recruited for qualitative interviews. Although the PCPs recognized kidney disease as an important comorbidity in T2D patients, testing for kidney disease was not consistently top of mind, with 56% reportedly performing kidney function testing in their T2D patients. PCPs most frequently reported using estimated glomerular filtration rate (eGFR) alone to monitor and stage DKD; only 25% PCPs reported testing for albuminuria. Most PCPs incorrectly believed that a majority of DKD patients are diagnosed in early stages. Also, early stages of DKD emerged as ambiguous areas of decision-making, wherein treatments prescribed greatly varied among PCPs. Lastly, early and accurate risk stratification of DKD patients emerged as the most important unmet need; which, if it could be overcome, was consistently identified by PCPs as a key to monitoring, appropriate nephrologist referrals, and intervening to improve outcomes in patients with DKD.

**Conclusions:**

Our study highlights important unmet needs in T2D DKD testing, staging, and stratification in the PCP setting that limit effective patient care. Health systems and insurers in the U.S. should prioritize the review and approval of new strategies that can improve DKD staging and risk stratification.

**Supplementary Information:**

The online version contains supplementary material available at 10.1186/s12889-021-10959-7.

## Introduction

An estimated 37 million people representing 15% of the total United States (U.S.) adult population have chronic kidney disease (CKD), which is identified by serum creatinine measurement as a test of kidney function to estimate glomerular filtration rate and a urine test for albuminuria, which is indicative of kidney damage in the glomerulus [1, 2]. Of these CKD patients, approximately 12 million (32%) have diabetic kidney disease (DKD), which includes any CKD with diabetes such as diabetic nephropathy and other causes of kidney diseases that may be distinguished by a kidney biopsy [3]. CKD in diabetes patients is a chronic, progressive disease and if undetected and untreated, leads to adverse clinical outcomes, such as end-stage kidney disease (ESKD), hyperkalemia, cardiac arrhythmias, cardiovascular morbidity, and mortality [4–6].

Additionally, CKD in diabetic patients results in a substantial incremental economic burden on patients and the healthcare system, with costs increasing exponentially with each advancing stage. Approximately 14% of all Medicare spending for patients aged 65 and older in 2017 was for diabetes with CKD [7]. Further, the costs for the diabetes population with CKD in 2017 were 51% higher than for those with diabetes but without CKD [7]. Moreover, the costs increased incrementally with advancing stage of CKD based on reduction in eGFR, with greatest expenditure for patients in ESKD. A retrospective analysis by Golestaneh et al. (2017) of 106,050 CKD patients showed that in the commercial population (aged < 65 years) average annual costs per patient were $16,770 in CKD stage G2 and $76,969 in CKD stage G4–5 [8]. In the Medicare population (aged ≥65 years) average annual costs per patient were $14,493 at CKD stage G2 and $46,128 at CKD stage G4–5 [8]. In the same study the per-patient average annual costs in ESKD patients (excluding cost of dialysis) were $121,948 and $87,339 in the commercial population and Medicare population, respectively. The financial burden imposed by CKD and ESKD results in estimated cost of $120 billion annually, which represents approximately 7.2% of the overall Medicare- fee-for-service reimbursed claims [7].

There has been an increase in the estimated prevalence of early stage CKD in the U.S. from 20 million in 1999 to 24 to 28 million in 2004, with several millions more at risk [9]. Given the enormous clinical and economic burden of CKD, early detection and intervention is critical to slow disease progression, prevent adverse clinical outcomes, and contain health care spending [9, 10]. Despite this recommendation from several organizations like KDIGO, National Kidney Foundation (NKF), and National Institutes of Health (NIH), studies show that in the U.S., only 10% of adults with CKD and 50% of adults whose kidney function is seriously impaired are even aware that they have the disease [1]. To facilitate timely diagnosis, clinical practice guidelines recommend annual screening of all type 2 diabetes patients using urine testing for albuminuria-creatinine ratio (uACR) and blood testing for serum creatinine to calculate eGFR [2, 11]. Although these kidney function tests are widely available and inexpensive, less than 50% of all patients with diabetes annually receive both tests [12]. The United States Renal Data System 2018 annual report noted that among Medicare beneficiaries with diabetes, only 41.8% had uACR testing in 2016 [7]. Although this proportion has increased from 2006 (26.4%), it is still markedly low, with fewer than half of the beneficiaries receiving basic guideline-recommended testing [7]. In an attempt to improve monitoring and diagnosis of CKD, the NKF in a partnership with the National Committee for Quality Assurance (NCQA), recently introduced the inclusion of the 'Kidney Health Evaluation for Patients with Diabetes' measure into the Healthcare Effectiveness Data and Information Set (HEDIS) [12]. This new performance measure assessing the percentage of adults aged 18–85 years with diabetes who have received both blood and urine CKD tests within the 12 months measurement interval, first measured in 2020, will be reported on by payers in 2021 [13]. As a majority of DKD patients receive medical care in the primary care setting, primary care provider (PCP) visits represent an important opportunity for diabetes patients to learn about DKD and manage their risk for onset and progression of the disease. However, evidence suggests that DKD is not optimally diagnosed or managed in diabetes patients receiving routine treatment in the primary care setting [14]. Therefore, the present study was undertaken to identify gaps in care and unmet needs in the diagnosis and monitoring of DKD in type 2 diabetes patients in the primary care setting using a series of focused qualitative interviews with PCPs.

## Methods

### Study design

This was a qualitative analysis of structured interviews with PCPs treating DKD patients. All participants provided written or verbal consent to participate in the study and for interviews to be audio-recorded. The study was reviewed by an independent Institutional Review Board (IRB) and exempted from full review due to its qualitative design resulting in no potential risk to patients.

### Participant recruitment

PCPs were recruited using an external recruiting agency (WebMD Medscape) that enlists panels of medical specialists for primary research. The recruiting agency recruits from a comprehensive list of physicians who have previously opted to participate in primary care research studies. The inclusion criteria, which were implemented through a 4-item screener questionnaire to confirm eligibility to participate included:

1. Currently board-certified in internal medicine or family medicine
2. Currently in clinical practice for more than 3 and less than 30 years
3. Spending most of their time (> 50%) in direct clinical care of patients compared to research or academic appointments
4. Providing care for greater than 3 individuals with type 2 diabetes patients in a week (on average)

Approximately 1800 invitations were sent out via email, and 138 responding PCPs were qualified to participate in the study based on entry criteria. Of these, 16 PCPs were selected based on approximately equal distribution of setting of care while maximizing the number of type 2 diabetes patients they saw per week (combination of purposive and random sampling). PCPs were selected such that there was a relatively equal distribution of specialty, settings of care, and patient volume. Recruitment was conducted by email and participants were briefed on the study purpose before interviews were scheduled. Repeat interviews were not allowed. PCPs were offered an honorarium of $300 upon completion of the interview which was planned to require 30–45 min.

### Interview process and interview guide

The research team developed a standardized (14-item) structured interview guide. Qualitative data were gathered through moderated telephone interviews conducted by 2 members of the research team (MD, SR) both of whom had prior training in qualitative research methodology and the specific use of the structured interview guide used in this research. The duration of the interviews ranged from 30 to 45 min. The structured interview guide consisted of a total of 14 questions that explored the background of PCPs (2 items), perceptions on current approaches to monitoring and managing type 2 diabetes patients (10 items), and perceptions of unmet needs and challenges in diagnosing and managing DKD patients (2 items). The structured interview guide used in this study is presented in Supplementary Table 1.

### Data collection and analysis

All interviews were digitally audio-recorded, stored in MP3 format and transcribed verbatim. The transcripts were not returned to participants for comment. The recruiting company anonymized the PCPs, such that the researchers did not have access to any personal or protected health information. The data transcripts were stored and analyzed in Microsoft Excel.

## Results

Characteristics of the 16 PCPs who participated in the study are provided in Table 1. Participating PCPs specialized in internal medicine (*n* = 8, 50%) or family medicine (*n* = 8, 50%) and worked in different settings of care including private practice (*n* = 6, 37.5%), academic medical center (*n* = 5, 31.25%) and integrated delivery network (*n* = 5, 31.25%).

**Table 1:** Characteristics of primary care physicians (*N* = 16)

Participant characteristics	N (%)
Medical specialty	
Internal Medicine	8 (50%)
Family Medicine	8 (50%)
Practice settings	
Private Practice	6 (37.5%)
Academic Medical Center	5 (31.25%)
Integrated Delivery Network	5 (31.25%)
Percent time spent in patient care	
≥ 80	15 (93.75%)
< 80	1 (6.25%)
Estimated Number of patients treated per week	
≤ 100	8 (50%)
> 100	8 (50%)

The qualitative interviews specifically sought to identify unmet needs in the management of DKD patients from the PCPs’ perspective. The transcripts of responses from participants were analyzed to identify four major themes:

1. Screening practices for kidney disease in type 2 diabetes patients
2. Gaps in PCP perceived knowledge that may contribute to underdiagnosis and inaccurate staging of DKD
3. Treatment variability and patient adherence by stage of disease
4. Assessment of the importance of risk stratification for optimal patient management and clinical outcomes

### Theme 1: Screening practices for kidney disease in type 2 diabetes patients

**Fig. 1:**
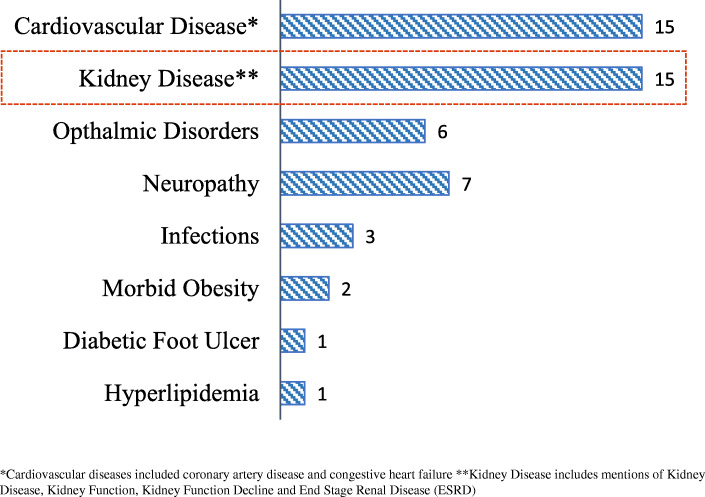
Most important and difficult to manage co-morbidities

Although PCPs recognized kidney disease as an important comorbidity in type 2 diabetes patients, testing for kidney disease was not top of mind for many PCPs. When specifically asked about kidney disease, 15/16 (94%) of PCPs mentioned it as a comorbidity that is difficult to manage in type 2 diabetes patients as shown in Fig. 1. However, when asked unprompted, 9/16 (56%) of PCPs reported performing kidney function testing in their patients, while 7/16 (44%) did not mention kidney function measures (Table 2). Kidney disease was mentioned as one of the first two areas of testing as part of the standard of care workup in type 2 diabetes patients by 2/9 (22%) of PCPs who reported performing kidney function testing in their patients and 2/16 (12.5%) of the total respondents.

**Table 2:**
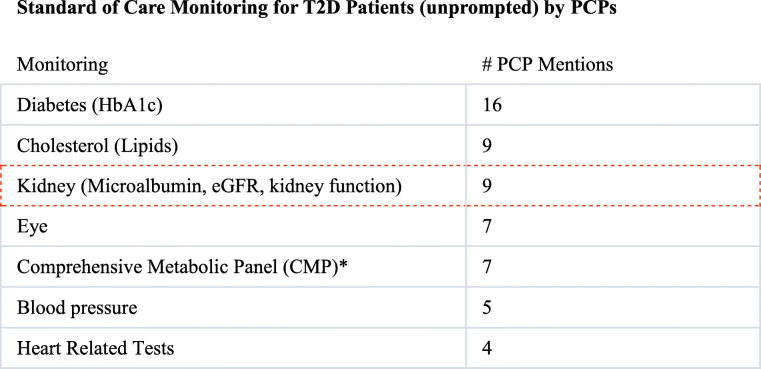
Standard of care tests in type 2 diabetes patients

^*^CMP includes glucose, calcium, sodium, potassium, CO_2_, chloride, albumin, protein, ALP, ALT, AST, bilirubin, BUN, creatinine

**Fig. 2:**
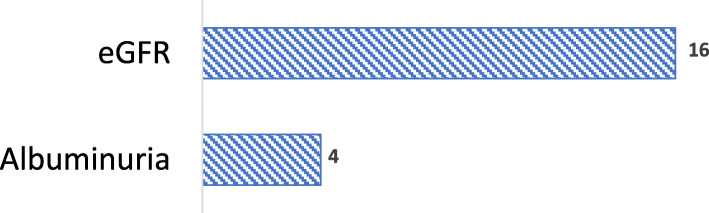
Metrics used for monitoring and staging of kidney disease

Further, although clinical practice guidelines recommend testing for both eGFR and uACR, which are both required to accurately monitor and stage kidney disease, PCPs most frequently reported using eGFR alone to monitor and stage DKD as shown in Fig. 2. In our study, 16/16 participants reported using eGFR to monitor and stage kidney disease, but only 4/16 (25%) reported testing for albuminuria. PCPs specified that both urine albumin and eGFR were monitored at least once per year.

### Theme 2: Gaps in PCP perceived knowledge that may contribute to underdiagnosis and inaccurate staging of DKD

**Fig. 3:**
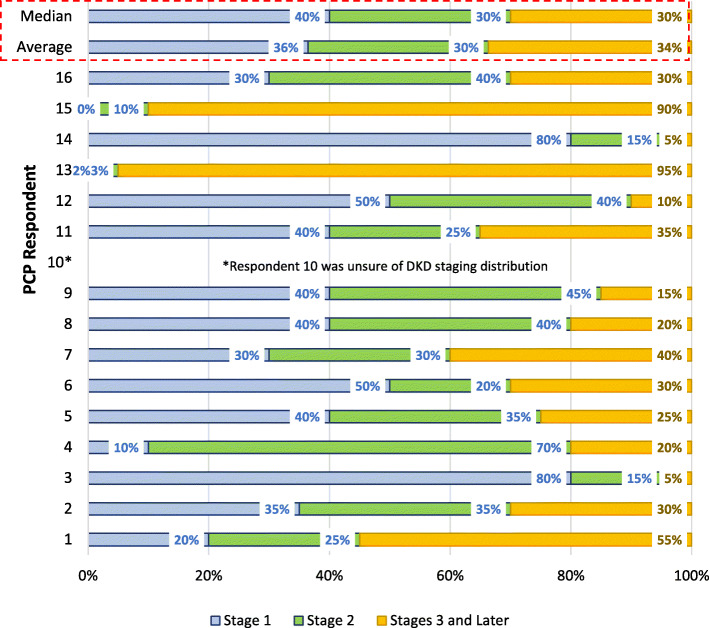
DKD stage distribution at stage according to PCPs

Most PCPs (13/15, 87%) in our study believed that the majority of DKD patients are diagnosed at stage G1 or G2 as opposed to stage G3 or later based on KDIGO staging guidelines [2]. According to the PCPs, on average 36% of diabetes patients are diagnosed with CKD at stage G1, 30% at stage G2, and 34% at stage G3 or later (Fig. 3). This belief is supported by the perception that most DKD patients regularly visit their PCP and, therefore, will be identified early during the disease. Furthermore, PCPs believe that diagnosis of DKD occurs in the later stage (stage G3 or more) only in new patients who have not visited a PCP for a long time, in patients with low medication compliance and/or patients with several uncontrolled comorbidities. One PCP commented,

*“Patients are diagnosed at stages 3 or later because they probably haven’t seen a physician in 10 years, had not had insurance, or they decided they were doing okay and stopped all medications. They normally are not showing up for the first time to my office with diabetic kidney disease in stage 3 (or higher)”.*

### Theme 3: Treatment variability and patient compliance by stage of disease

Treatment for DKD is dependent on stage of the disease. PCPs mentioned that treatments for CKD stages G1 and G2 in diabetes patients are mostly focused on lifestyle modification, patient education, nutritionist referrals, and avoidance of medications that can be harmful to the kidneys such as non-steroidal anti-inflammatory drugs (NSAIDs). Such management strategies aim to slow kidney disease progression and restore kidney function. PCPs in our study reported that diabetic patients in CKD stage G1 and G2 typically visit PCPs every 3 to 6 months. They mentioned that they begin to focus on pharmaceutical interventions in diabetes patients at CKD stage G3 and more severe onwards, with follow-up PCP visits in this stage reported as usually occurring approximately every 3 months. At stage G4, patients are typically already referred to a nephrologist, with PCPs continuing to manage the patient except their kidney disease, which is managed by the nephrologist. PCPs stated that at stage G5, the patient is put on dialysis and/or the kidney transplant wait list. Table 3 outlines the monitoring and treatment protocol reported by PCPs at each stage of CKD in our interviews. There was consensus among PCPs in our study regarding this stage-specific protocol. Further there is consistency in PCPs incorrectly suggesting that at stage G1 and G2, treatment protocols are ambiguous because patients are not considered at risk of kidney function decline in these early stages. This belief is contradicted by published literature [2, 15–17].

**Table 3:** Monitoring and treatment protocol by stage of DKD

Stage	Treatment Protocols	PCP Visit Frequency
**Stage 1**	• Monitor, recommend lifestyle modifications	Every 3 to 6 months
	• Patient education	
	• Avoid NSAIDs and other medications that can be harmful to kidneys	
	• Introduce some pharmaceutical treatment	
**Stage 2**	• Consider different pharmaceutical treatments	Every 3 to 6 months
	• Refer to nutritionist	
	• Increase frequency of visits to every 3 months	
	• Consider initial referral to nephrologist	
**Stage 3a**	• Monitor calcium phosphate metabolism and iron stores	Every 3 months
	• Focus more on pharmaceutical treatment	
	• Referral to nephrologist	
**Stage 3b**	• Refer to nephrologist for regular visits	Every 3 months
**Stage 4**	• Kidney-related decisions are made by the nephrologist	N/A
	• Still managing patient, but not their kidney condition	
**Stage 5 (ESKD)**	• Still managing patient when hospitalized, but not their kidney condition	N/A

Moreover, lack of patient adherence to these treatment protocols was identified as a key unmet need in DKD management. PCPs noted that patient compliance to lifestyle recommendations or treatment protocols is a key component of effective management of DKD patients; however, they noted that the lack of adherence to lifestyle behavior modifications (e.g., diet and exercise), and treatment regimens can increase patients’ risk of progression, morbidity, hospitalizations and mortality. PCPs believed that accurate and early identification of the risk of progression of DKD could increase patient compliance in this population.

### Theme 4: Assessment of the importance of risk stratification for optimal patient management and clinical outcomes

All 16 PCPs (100%) mentioned that accurate risk assessment methods can help predict the risk of disease progression that identifies the need for treatment changes, monitoring of response to medications, optimized modification of medications and dosages, and earlier decision-making about the optimal time to refer a patient to a nephrologist. They also acknowledged that inaccurate risk stratification can negatively impact treatment management and patient compliance and outcomes. Several physicians commented on the potential of suboptimal or contraindicated therapies being prescribed to patients as a result of inaccurate risk stratification as exemplified by the following quote:

*“[If risk stratification is inaccurate and] if an orthopedist recommends diclofenac, which is prescription anti-inflammatory NSAID, catastrophic and dangerous things can happen. You could end up worsening their kidney failure.”*

Furthermore, PCPs indicated that inaccurate risk stratification can also result in suboptimal therapy, which can lead to rapid progression, increased hospital inpatient admissions, emergency room visits, potential kidney failure, need for dialysis, decreased quality of life, and increased costs. One PCP noted that inaccurate DKD staging will increase morbidity, mortality and the number of patients ending up in the Emergency Room (ER):

*“[If DKD is inaccurately staged] the patient will fall sick faster. They may end up in the ER. Morbidity and mortality can increase”.*

PCPs also mentioned that currently there are no tests that can accurately predict the risk of CKD progression in diabetes patients, which in turn delays optimal CKD management. One physician explained: “*I wish there was a better way to identify risk for kidney function decline earlier, before their kidney disease progresses to stage two and three*.”

## Discussion

This qualitative study conducted among 16 PCPs documents several substantial gaps in care and unmet needs in monitoring and management of DKD patients receiving routine treatment in the primary care setting. First, guideline-recommended kidney function tests are not routinely and adequately performed in type 2 diabetes patients. Second, many PCPs claimed that most DKD patients are diagnosed in early stages of the disease. Third, they believed that treatment varies by stage and is ambiguous at early stages, which inhibits or delays optimal medical management. Finally, all PCPs felt that early and accurate risk stratification is a critical unmet need in effective management of DKD patients, and if addressed, can increase patient compliance, improve patient outcomes, and reduce healthcare costs.

Our study found that compared to other screening tests commonly used in diabetes patients, such as HbA1c, kidney function testing has a lower priority among PCPs even though they acknowledge that monitoring represents an opportunity to identify DKD early and slow disease progression through improved medical intervention. This is consistent with prior literature reporting low rates of kidney function testing in diabetes patients [7, 18]. A retrospective analysis conducted on data from a Laboratory Corporation of America regional laboratory in Charlotte, NC found that only 36.2% of patients who had diagnosed chronic kidney disease underwent creatinine testing [18]. In high risk patients with CKD and CVD, these rates were found to be even lower [18]. This trend of inconsistent kidney function testing in diabetes patients may be attributable to lack of available time and resources in the primary care setting, and the complexity of managing diabetes patients [19]. Alerts through electronic health records (EHRs) may address some of these barriers; however, studies show that the EHR data may not always be adequately utilized by practicing physicians for monitoring and management purposes [19, 20]. Our study also found that while physicians report regularly monitoring eGFR, albuminuria is inconsistently assessed in diabetes patients managed by PCPs. According to the KDIGO and ADA clinical practice guidelines, both eGFR and uACR are independent and necessary to identify and stage kidney disease in type 2 diabetes patients, as well as provide an approximation for the risk of progression [2]. Detection of albuminuria also allows earlier intervention as it can assess early DKD before the eGFR drops below 60 mL/min/1.73 m^2^_,_ which is the eGFR-based threshold for the definition of CKD [21]. Moreover, albuminuria testing, specifically microalbuminuria, is important as it is an independent risk factor for cardiovascular disease [22]. Patients with CKD are at increased risk for subsequent CVD even at early stages, and PCPs need to undertake effective management strategies that reduce this risk.

The perception of PCPs who participated in our study that most patients (66%) are diagnosed with DKD in early stages (stages G1–2) is not consistent with data from previous studies, which demonstrated that identification of CKD by PCPs is low. A prior study reported that only 12% of patients with laboratory-based diagnosis of CKD were identified as having CKD by their physicians [17]. Awareness remains low during the early stage of CKD, and diagnosis of CKD typically occurs during later stages of the disease [1, 23]. Only 1 in 2 patients with very low kidney function who are not on dialysis know that they have CKD [1]. A multi-center, observational study of 9307 adults with type 2 diabetes by Szczech et al. 2014 showed that of the 5036 patients with CKD, the proportion of patients correctly identified as having CKD was: 1.1% in stage G1, 4.9% in stage G2, 18% in stage G3, 52.9% in stage G4 and 58.8% in stage 5 [23]. In addition, PCPs believe that treatment ambiguity in earlier stages and patient non-compliance can pose barriers to DKD management. These findings corroborate results from a qualitative study by Sperati et al. (2019), in which PCPs identified limited familiarity with guidelines, difficulty managing risk factors associated with disease progression, and the belief that CKD is not reversible as major barriers in providing optimal CKD care [24]. Such challenges may be further exacerbated by the ambiguous nature of the treatment landscape. Similarly, patient non-compliance may be driven by lack of understanding of the severity of kidney disease, the clinical implications for their long-term health and quality of life, and high out-of-pocket costs associated with chronic kidney disease [24, 25].

Lastly, PCPs believe that accurate risk stratification is a critical unmet need in the DKD population delaying early intervention and effective management of DKD. Several prior publications support this claim and note that accurate risk stratification in kidney disease is important for improving the health and wellness of patients with this disease [16, 26]. Our research findings build on existing evidence to provide a detailed view of the gap in evaluation and management of DKD patients in the primary care setting. These findings provide valuable insights that can be used to improve DKD care delivery in the U.S.

There were several limitations to this study. First, the study was conducted in a small sample of 16 PCPs, which limits the generalizability of findings. Second, this study consisted of PCPs who self-selected to participate in the study, which could potentially introduce selection-bias. The eligible participants who agreed to enter the study may differ from those who refused to participate or were challenging to reach. For instance, participants who entered the study had ready access to a computer and/or internet facilities compared to those who did not enter, leading to inherent differences. However, given the extensive outreach through electronic recruiting having substantial time and cost implications, this was our preferred approach. Third, the data in this study is based on clinicians’ self-report of existing knowledge and practice processes. We acknowledge biases that can arise from social desirability, recall period, or selective recall as a result of self-report [27]. Future studies should assess the concordance between self-report data found in our study and objective data from EHRs. Finally, the potential gap between reported knowledge of PCPs and actual practice patterns is worth acknowledging. However, despite these potential limitations, we believe that these contemporary findings represent important themes reported in prior research and highlight unmet needs for care and management of DKD patients.

## Conclusion

Our study highlights the unmet needs related to early detection, staging, and prevention of DKD. For successful DKD management in the type 2 diabetic population, interventions to facilitate guideline-concordant care, accurate risk stratification tools, and patient and physician education are required. Such efforts can facilitate guideline adherence, result in timely referral to nephrologists, improve clinical outcomes, and reduce the rate of progression from DKD to ESKD, thereby reducing healthcare costs in these patients.

## Supplementary Information

**Additional file 1: Table 1.** Summary of interview guide.

## Data Availability

The data collected through primary interviews used and/or analyzed during the current study are available from the corresponding author on reasonable request.

## Abbreviations

CKD: Chronic kidney disease
DKD: Diabetic kidney disease
eGFR: Estimated glomerular filtration rate
EHR: Electronic health records
ESKD: End-stage kidney disease
HEDIS: Healthcare Effectiveness Data and Information Set
IRB: Institutional Review Board
NCQA: National Committee for Quality Assurance
NKF: National Kidney Foundation
PCP: Primary Care Physician
T2D: Type 2 diabetes
uACR: Albuminuria-creatinine ratio
