# Supplementary material for: A qualitative study documenting unmet needs in the management of diabetic kidney disease (DKD) in the primary care setting
Source: BMC Public Health. 2021 May 17;21:930. doi: 10.1186/s12889-021-10959-7 (PMC8127260; doi:10.1186/s12889-021-10959-7)
Supplement: Supplementary file 1 — Additional file 1: Table 1. Summary of interview guide. [file 12889_2021_10959_MOESM1_ESM.docx]

**A Qualitative Study Documenting Unmet Needs in the Management of Diabetic Kidney Disease (DKD) in the Primary Care Setting**

**Manasi Datar,** PhD^1^, **Saranya Ramakrishnan**, MPH^1^, **Elizabeth Montgomery**^2^, **Steven G. Coca**, D.O., M.S.^3^, **Joseph A. Vassalotti**, MD^2,3^, and **Thomas Goss**, PharmD^1*^
*Corresponding author

^1^Boston Healthcare Associates

^2^National Kidney Foundation, Inc., New York, NY

^3^Icahn School of Medicine at Mount Sinai, New York, NY

**SUPPLEMENTARY MATERIAL**

| **Table 1** Summary interview guide | |
| --- | --- |
| **Background** | |
|  | 1. Please describe your practice    1. Probe: Type of practice, hospital affiliation, number of patients, patient demographics, practice size |
|  | 1. Do you keep up to date with guideline recommendations? How do you do this? |
| **Current practice** | |
|  | 1. What are your perspectives on current approaches to monitoring and managing type 2 diabetes patients?    1. Which specific tests/procedures/exams are used? |
|  | 1. What are the comorbidities/complications of greatest concern when managing patients with type 2 diabetes?    1. How difficult are they to screen for and manage?    2. Please rank the aforementioned comorbidities/complications in order of ‘most important to address’ to ‘least important to address’. |
|  | 1. How actively do you monitor/manage the kidney-related complications associated with diabetes?    1. Is this something that PCPs mostly manage or is it the nephrologists? If so, at what point do you refer?    2. What criteria do you routinely use to help in deciding when you would refer a patient to a nephrologist for a consultation or to manage the patient’s kidney disease?    3. What tests are used in monitoring kidney disease? Probes: eGFR, Albuminuria |
|  | 1. Do you use any algorithms/nomograms to assess severity/staging of Diabetic Kidney Disease? If yes, which algorithms/nomograms? Probes: KDIGO classification    1. On a scale of 1-5, with one being very easy and 5 being very complex, can you comment on the complexity of the algorithm    2. If no,       1. Is the KDIGO classification used for this purpose?       2. If yes, on a scale of 1-5, with one being very easy and 5 being very complex, can you comment on the complexity of the algorithm       3. If not, why not? Probe: Is it because you rely on referral? |
|  | 1. For each stage of DKD what percentage of patients are initially diagnosed at this stage? Probes: stage 1, stage 2, stage 3a, stage 3b, stage 4, stage 5 (ESRD)    1. What are the factors that result in delayed diagnosis (stage 3 or above)? |
|  | 1. In your experience over the past 1-2 years, how frequently have you had a patient decline rapidly (or crash) suddenly, acutely requiring dialysis before there was time for planning how they would manage dialysis/transplant? |
|  | 1. What is the importance of DKD risk stratification or staging?    1. Is this done for all type 2 diabetes patients or on a case-by-case basis? If case-by-case, in which patients?    2. How do you use this information in DKD management?    3. What are the advantages of tests used for DKD identification and stratification? Are they reliable?    4. What are the disadvantages? |
|  | 1. Are patients in different DKD stages treated differently? What are the stage-specific treatment protocols for stage 1, stage 2, stage 3a, stage 3b, stage 4, and stage 5 |
|  | 1. Are there any guidelines you follow for the management of DKD? If yes, which ones?    1. [If yes] Please describe the guidelines    2. [if yes] Do you adapt or modify guidelines based on the population you serve? If so, are these adaptations generally more comprehensive or less comprehensive than specialty society guidelines? |
|  | 1. Are improvements in DKD clinical care connected with improved financial performance on risk-based contracts? |
| Unmet needs | |
|  | 1. What are the current unmet needs/challenges in DKD risk stratification? |
|  | 1. What happens if DKD risk stratification is not accurate?    1. How does it impact patient management?    2. How does it impact patient outcomes? |
